# Uterine mesenchymal tumour with a novel *EWSR1::CTBP1* gene fusion

**DOI:** 10.1007/s00428-025-04205-3

**Published:** 2025-10-11

**Authors:** Beata Bode-Lesniewska, Frank Illigen, Matthias S. Matter

**Affiliations:** 1 Institute of Pathology, Cantonal Hospital Lucerne, Lucerne and University of Zurich, CH-6000 Lucerne, Switzerland; 2 Gynecology, Zug, Switzerland; 3 Institute of Medical Genetics and Pathology, University Hospital of Basel, Basel, Switzerland

**Keywords:** Mesenchymal tumours, Uterus, FISH, Next-generation sequencing

## Abstract

A growing number of mesenchymal uterine tumours are defined by a specific molecular aberration. We present the case of a 65-year-old woman who presented with postmenopausal bleeding and a large intramural uterine tumour. Histopathological analysis of the resected uterus revealed an intramural, mesenchymal neoplasm comprising of spindle and epithelioid cells, with no immunohistochemical expression of lineage-specific markers and low proliferative activity. Molecular testing using next-generation sequencing (NGS) revealed an *EWSR1::CTBP1* gene fusion, which was confirmed by the presence of *EWSR1* gene rearrangement detected using fluorescence in situ hybridisation (FISH). Staging revealed no further tumour manifestations, and the 3-year follow-up was uneventful. The *EWSR1::CTBP1* gene fusion has never previously been reported in uterine tumours, having been reported in the literature only once, in the context of a gastroblastoma. The presented case expands the range of the gene-fusion-associated mesenchymal tumours of the uterus.

**Supplementary Information:**

The online version contains supplementary material available at 10.1007/s00428-025-04205-3.

## Introduction

The vast majority of the mesenchymal tumours in the uterine corpus [8] are smooth muscle neoplasms, ranging from common benign leiomyomas with their numerous morphological variants to rare leiomyosarcomas. The smooth muscle differentiation of these neoplasms is obvious in conventional slides and can by confirmed immunohistochemically with positive reaction for smooth muscle actin (SMA) and desmin. While the smooth muscle tumours lack reproducible defining genetic alterations except for fumarate hydratase-deficient leiomyoma, endometrial stromal neoplasms carry in most cases typical gene fusions. The most common are *JAZF1::SUZ12* in low-grade stromal sarcomas (LGESS) and *YWHAE::NUTM2A/B* or *BCOR::ZC3H7B* (or other rarer variants) in high-grade endometrial sarcomas (HGESS). The advent of regular genetic testing [1, 5, 10] has led to the identification of several other reproducible aberrations in uterine mesenchymal tumours. This has resulted in a precise classification, such as the identification of *ALK1* gene rearrangements in the inflammatory myofibroblastic tumours (IMTs), SMARCA4 deficiency in SMARCA4 deficient uterus sarcomas and rearrangements of *ESR1* or *GREB1* genes in uterine tumours resembling ovarian sex cord tumours. Rare examples of intrauterine solitary fibrous tumour, NTRK sarcomas or alveolar soft part sarcomas may also be encountered as primary tumours of the uterus. The term of undifferentiated uterine sarcoma (UUS) is used as the diagnosis of exclusion for a small group of mostly aggressive sarcomatous tumours that do not fulfil the criteria for other entities. Before a diagnosis of a primary uterine mesenchymal tumour is made, any form of sarcomatous overgrowth in carcinosarcomas or adenosarcomas must be excluded.

In this manuscript, we describe an unusual, low-grade mesenchymal tumour of the uterine corpus in a 65-year-old woman with a unique gene translocation *EWSR1::CTBP1,* which was detected by NGS and confirmed by FISH. The *EWSR1::CTBP1* translocation has only been previously described once, in a case of gastroblastoma of the stomach [6, 7] and has not yet been reported in the uterine tumours. The long follow-up of 36 months without further tumour manifestations suggests benign or low-grade clinical behaviour.

## Case description, morphological and immunohistochemical analysis

**Fig. 1:**
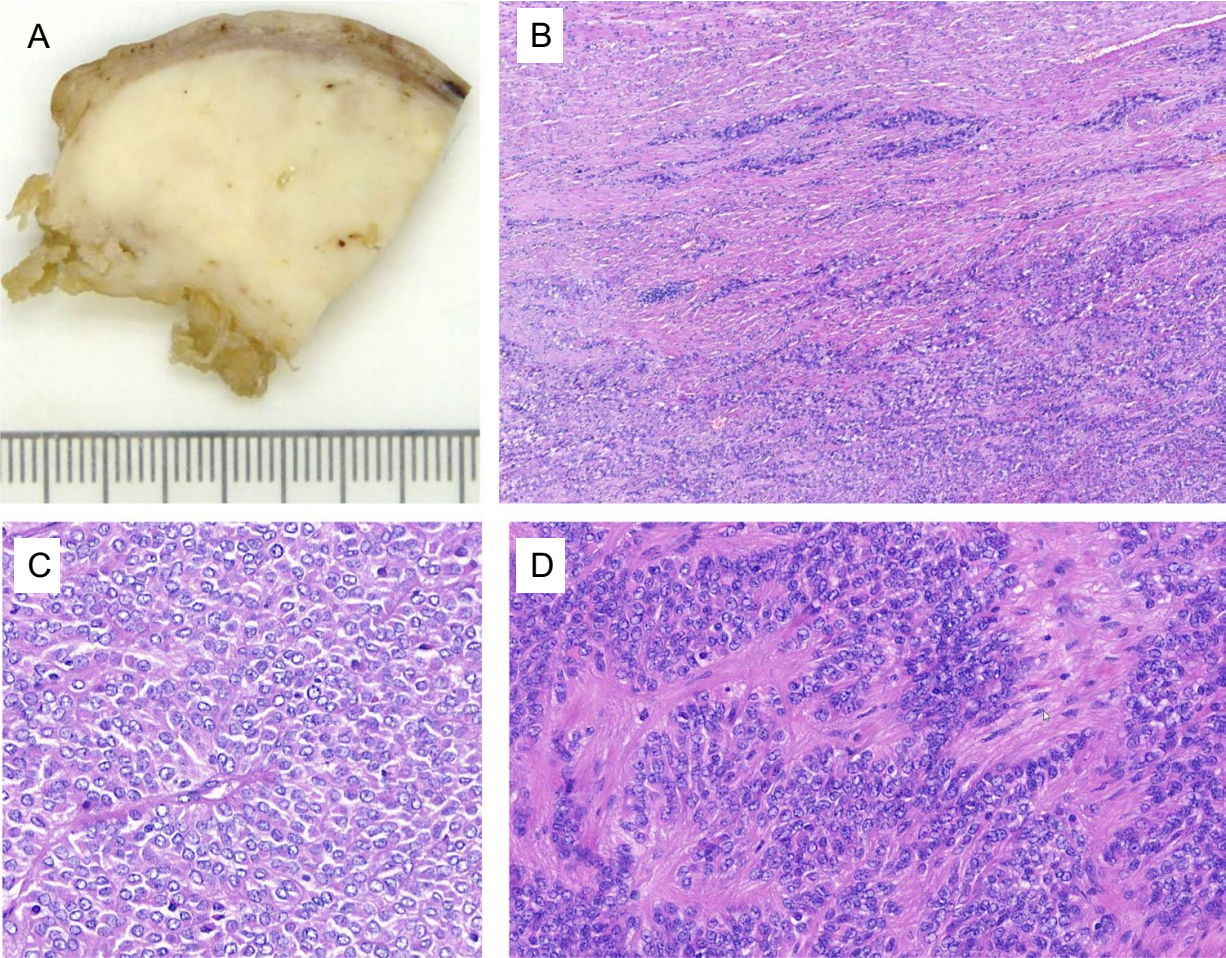
Histology of the uterine tumour: **A** cut section of one of the tumour fragments. **B** Cellular neoplasia with infiltrative peripheral growth. **C** Large areas of the tumour with epithelioid aspect and large nuclei with prominent nucleoli and excentric eosinophilic cytoplasm. **D** Trabecular areas displaying fibrous septa

A 65-year-old woman presented with postmenopausal bleeding. The patient’s relevant past medical history was unremarkable except for a remote history of conisation due to HSIL/CIN III. The current curettage specimen showed no signs of malignancy. A laparoscopic bilateral adnexectomy and hysterectomy with morcellation of the uterus was performed. On gross examination, the uterine fragments consisted of a separated cervix and a large portion of the corpus containing an intramural, well-delineated, solid, whitish tumour mass (Fig. 1A), as well as several additional tumour fragments, with a total weight of 115 g. The reconstructed size of the large intramural tumour was at least 7.0 cm. Several small (max 1.0 cm) typical intramural leiomyomas were present. The endometrium and both adnexa showed macroscopically and microscopically no relevant findings.

Histologically (Fig. 1B–D), the myometrial mass showed areas of solid spindle cell and epithelioid neoplasia with trabecular growth. A fibrotic background was focally present, but no production of a differentiated extracellular matrix was observed. The tumour cells were of medium size, rather monomorphic, with broad eosinophilic cytoplasmic rims and distinct cell borders. The oval to roundish, vesicular nuclei with coarse and irregular chromatin showed practically no mitotic activity. At the periphery of the tumour infiltrative growth pattern with no capsule could be seen. There was no tumour necrosis.

**Fig. 2:**
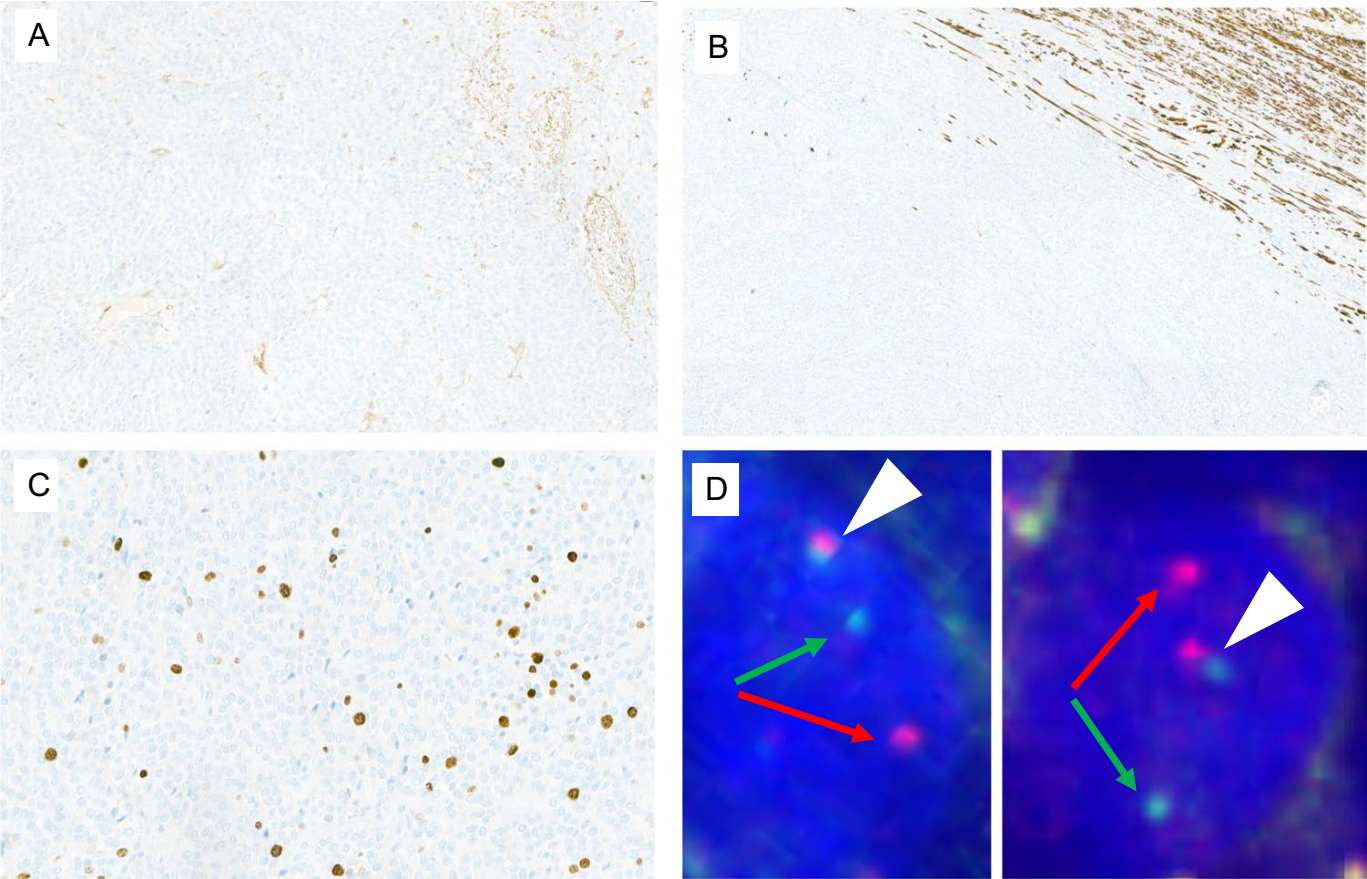
Immunohistochemistry and FISH: **A** SMA reaction highlights the perivascular structures, while tumour cells remain negative. **B** Desmin is negative in the neoplastic cells, which at the periphery intermingle with smooth muscle cells of the myometrium. **C** Low proliferation index of less than 10% by Ki67 immunohistochemistry. **D** FISH with a break-apart probe of the *EWSR1* gene: one fused signal (white arrowhead) and one separate red and green signal (red/green arrows) per nucleus indicating the rearrangement of the *EWSR1* gene

Extensive immunohistochemical analysis revealed negativity for epithelial (AE1/AE3, EMA and CK7), myogenic (SMA and desmin) (Fig. 2A–B) and neural/melanocytic (S100, SOX10 and HMB45) markers, as well as for hormone receptors, WT1, PAX8, CD10, inhibin, synaptophysin, CD56, ALK1, BRAF and NTRK. Expression of fumarate hydratase as well as nuclear expression of INI1 was retained. The proliferation index Ki67 was low of less than 10% (Fig. 2C). The FISH analysis (Fig. 2D) demonstrated the rearrangement of the *EWSR1* gene, which is consistent with the results of the NGS analysis as described below.

Staging was performed 3 months after the hysterectomy not revealing residual or recurrent tumour and not showing any signs of metastatic disease.

At last follow-up at 36 months following hysterectomy, there were no signs of local recurrence or metastatic disease.

## Genetical analysis (DNA sequencing)

Sample of the uterine tumour was analysed by parallel sequencing of DNA with the Oncomine™ Comprehensive Panel Version 3 (Thermo Fisher, USA) covering 135 genes (see supplementary for list of the genes covered) as well as the FusionPlex™ Sarcoma Panel (Archer, USA) covering 137 genes and their fusion partners. Tissue selection, DNA and RNA extraction were done using an in-house pipeline (see supplementary for details). Raw data was processed automatically on the Torrent Server™ and aligned to the reference hg19 genome. NGS data analysis was performed on Ion Reporter Analysis Software (ThermoFisher Scientific) using the AmpliSeq Colon-Lung single sample workflow. QC was performed manually for each sample based on the following metrics: number of reads per sample > 500,000 on-target reads > 90%, read uniformity > 90%, mean read coverage > 2000. The sequencing data of the QC passing samples was then uploaded in BAM format to the Ion Reporter™ Analysis Server for variant calling and annotation. Variants were filtered based on Phred score > 50, allele coverage > 500 and a minimum allele frequency of 2%. Furthermore, polymorphisms were filtered against UCSC common SNP, ExAC, 1000 Genome and 5000 Exome databases.

**Fig. 3:**
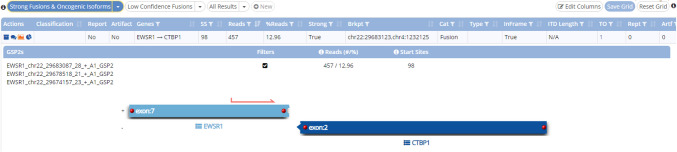
Structure of the gene fusion detected by next-generation sequencing (NGS): in frame fusion transcript between the exon 7 of the *EWSR1* gene and the exon 2 of the *CTBP1* gene (Exon 2) (*EWSR1* NM_005243.3, exon:7; *CTBP1* NM_001328.2, exon:2)

Sequencing analysis revealed no known pathogenic mutations or copy number changes. The in-frame fusion transcript between the exon 7 of the *EWSR1* gene and the exon 2 of the *CTBP1* gene (Exon 2) was found corresponding to the *EWSR1::CTBP1* gene fusion (*EWSR1* NM_005243.3, exon:7; *CTBP1* NM_001328.2, exon:2) (Fig. 3A).

## Discussion and conclusion

Mesenchymal uterine tumours are a diverse group of non-epithelial tumours, ranging from fully benign conventional leiomyomas to highly aggressive entities (leiomyosarcomas, high grade endometrial stromal sarcomas (HGESS) or undifferentiated uterine sarcomas) [8]. Growing knowledge has been accumulated on their genetic background, deepening our understanding of the pathogenesis and offering therapeutic options in selected cases (e.g. in *ALK1* or *NTRK* genes rearranged tumours). Genetic testing has become the standard analysis for microscopically or immunohistochemically unusual tumours not fitting the standard criteria of the defined entities. We describe an unusual, large, uterine intramural tumour in a 65-year-old patient with unspecific immunohistochemical properties, notably lacking the expression of myogenic markers. The tumour was found to harbour the *EWSR1::CTBP1* gene fusion as revealed by NGS, and the presence of the *EWSR1* gene rearrangement was confirmed by FISH.

The *EWSR1* gene is as a member of the TET/FET family and encodings the EWS protein, which plays an important role in the RNA processing, gene transcription and DNA repair. The *EWSR1* gene is involved in the pathogenesis of several, mostly, but not exclusively, mesenchymal tumours [15]. It forms fusions with many partner genes in numerous neoplasms, e.g. *FLI1* in Ewing sarcoma (ES) [3], *NR4A3* in extraskeletal mesenchymal chondrosarcoma (EMC) [13], *ATF1* or *CREB1* in clear cell sarcoma (CCS) or angiomatoid fibrous histiocytoma (AFH) [4, 16], *CREB3L2/1* in low grade fibromyxoid sarcoma/sclerosing epithelioid fibrosarcoma (LGFMS/SEF) [9, 11], *DDTI3* in myxoid liposarcoma (MLPS) [12] or variable fusion partners in myoepithelial neoplasms of soft tissue [14]. *EWSR1* gene is in many known fusions interchangeable with the *FUS* gene, another member of the TET/FET gene family.

The *CTBP1* gene codes for the C-terminal binding protein 1, which is a transcriptional corepressor, modulating transcriptional repression by recruiting chromatin-modifying proteins. Aberrations involving *CTBP1* gene are not well documented in the literature, despite the fact that CTBP1 overexpression has been observed in many different cancers, including cancers of the prostate, colon, breast and ovary, as well as melanoma and leukaemia [2].

To date, only one other case of a tumour carrying the *EWSR1::CTBP1* gene fusion has been reported in the literature [7]. The 6.3-cm tumour was located in the stomach wall of a 17-year old patient with Wiskott-Aldrich syndrome, showed biphasic morphology with the expression of cytokeratin and neuroendocrine markers and was classified as a gastroblastoma. The most common gastroblastoma *MALAT1::GLI1* gene fusion or an alternative *GLI1* gene fusion was not present. During the follow-up of 23 months no further manifestations of the completely resected tumour occurred. The uterine tumour described here differs significantly in its immunohistochemical properties, not showing biphasic pattern upon extensive sampling. Both the gastric and the uterine tumours followed a favourable clinical course, with no evidence of recurrent or metastatic disease after 2 or 3 years of follow-up, respectively.

In conclusion, we describe a unique uterine tumour characterised by the *EWSR1::CTBP1* gene fusion, which has not previously been reported in this location. This finding further expands the genetic landscape of uterine mesenchymal neoplasia. Apart from its large size of (7.0 cm) and infiltrative peripheral growth, there were no worrisome histopathological features (low mitotic activity, no pleomorphism and no necrosis). During the 36-month follow-up period, there were no signs of local recurrence or metastatic spread; however, the prognosis remains uncertain due to a lack of the reported comparative literature data.

## Supplementary Information

Below is the link to the electronic supplementary material.

ESM1 (DOCX 31.1 KB)
