# Supplementary material for: Uterine mesenchymal tumour with a novel EWSR1::CTBP1 gene fusion
Source: Virchows Arch. 2025 Oct 11;487(5):1137–41. doi: 10.1007/s00428-025-04205-3 (PMC12647275; doi:10.1007/s00428-025-04205-3)
Supplement: Supplementary file 1 — ESM1 (DOCX 31.1 KB) [file 428_2025_4205_MOESM1_ESM.docx]

Bode-Lesniewska, B., et al: **Uterine mesenchymal tumour with a novel *EWSR1::CTBP1* gene fusion**

**Supplementary**

**Genes covered by the sequencing panel**

Oncomine™ Comprehensive Panel v3 (hotspots of 87 genes covered):

*AKT1,AKT2,AKT3,ALK,AR,ARAF,AXL,BRAF,BTK,CBL,CCND1,CDK4,CDK6,CHEK2,CSF1R,CTNNB1,DDR2,EGFR,ERBB2,ERBB3,ERBB4,ERCC2,ESR1,EZH2,FGFR1,FGFR2,FGFR3,FGFR4,FLT3,FOXL2,GATA2,GNA11,GNAQ,GNAS,H3F3A,HIST1H3B,HNF1A,HRAS,IDH1,IDH2,JAK1,JAK2,JAK3,KDR,KIT,KNSTRN,KRAS,MAGOH,MAP2K1,MAP2K2,MAP2K4,MAPK1,MAX,MDM4,MED12,MET,MTOR,MYC,MYCN,MYD88,NFE2L2,NRAS,NTRK1,NTRK2,NTRK3,PDGFRA,PDGFRB,PIK3CA,PIK3CB,PPP2R1A,PTPN11,RAC1,RAF1,RET,RHEB,RHOA,ROS1,SF3B1,SMAD4,SMO,SPOP,SRC,STAT3,TERT,TOP1,U2AF1,XPO1.*

Oncomine™ Comprehensive Panel v3 (48 genes fully covered):

*ARID1A,ATM,ATR,ATRX,BAP1,BRCA1,BRCA2,CDK12,CDKN1B,CDKN2A,CDKN2B,CHEK1,CREBBP,FANCA,FANCD2,FANCI,FBXW7,MLH1,MRE11A,MSH2,MSH6,NBN,NF1,NF2,NOTCH1,NOTCH2,NOTCH3,PALB2,PIK3R1,PMS2,POLE,PTCH1,PTEN,RAD50,RAD51,RAD51B,RAD51C,RAD51D,RB1,RNF43,SETD2,SLX4,SMARCA4,SMARCB1,STK11,TP53,TSC1,TSC2.*

Oncomine™ Comprehensive Panel v3: copy number variation, 47 genes covered:

*AKT1,AKT2,AKT3,ALK,AR,AXL,BRAF,CCND1,CCND2,CCND3,CCNE1,CDK2,CDK4,CDK6,EGFR,ERBB2,ESR1,FGF19,FGF3,FGFR1,FGFR2,FGFR3,FGFR4,FLT3,IGF1R,KIT,KRAS,MDM2,MDM4,MET,MYC,MYCL,MYCN,NTRK1,NTRK2,NTRK3,PDGFRA,PDGFRB,PIK3CA,PIK3CB,PPARG,RICTOR,TERT.*

Archer™ Custom V2 Panel, covering fusions involving following genes:

*ACVR2A, AKT1, AKT2, AKT3, ALK, AR, ARHGAP26, ARHGAP6, AXL, BCOR, BRAF, BRD3, BRD4, CAMTA1, CCNB3, CCND1, CD274, CIC, CRTC1, CSF1, CSF1R, CTNNB1, DNAJB1, EGF, EGFR, EPC1, ERBB2, ERBB4, ERG, ESR1, ESRRA, ETV1, ETV4, ETV5, ETV6, EWSR1, FGF1, FGFR1, FGFR2, FGFR3, FGR, FOS, FOSB, FOXO1, FOXO4, FOXR2, FUS, GLI1, GRB7, HMGA2, HRAS, IDH1, IDH2, IGF1R, INSR, JAK2, JAK3, JAZF1, KIT, KRAS, MAML2, MAP2K1, MAST1, MAST2, MBTD1, MDM2, MEAF6, MET, MGEA5, MKL2, MN1, MSMB, MUSK, MYB, MYBL1, MYC, MYOD1, NCOA1, NCOA2, NCOA3, NFATC2, NFE2L2, NFIB, NOTCH1, NOTCH2, NR4A3, NRAS, NRG1, NTRK1, NTRK2, NTRK3, NUMBL, NUTM1, PAX3, PAX8, PDGFB, PDGFD, PDGFRA, PDGFRB, PHF1, PHKB, PIK3CA, PKN1, PLAG1, PPARG, PRDM10, PRKACA, PRKACB, PRKCA, PRKCB, PRKCD, PRKD1, PRKD2, PRKD3, RAD51B, RAF1, RELA, RET, ROS1, RSPO2, RSPO3, SS18, SS18L1, STAT6, TAF15, TCF12, TERT, TFE3, TFEB, TFG, THADA, TMPRSS2, USP6, VGLL2, WWTR1, YAP1, YWHAE.*

**Tissue selection, acid nucleic extraction**

For acid nucleic isolation, the area of interest was marked on H&E stained tissue slides and then macrodissected by scratching. The DNA was isolated using the Maxwell DNA purification kit according to the manufacturer’s protocol. The DNA concentration was quantified using the Qubit® dsDNA HS Assay Kit (ThermoFisher Scientific). The Ion AmpliSeq Library Kit V.2.0 (ThermoFisher Scientific) was used to prepare the libraries from 10-20 ng of DNA. The Ion Ampliseq HiFi Master Mix (ThermoFisher Scientific) was used to prepare the amplicons that were digested with FUPA reagent in order to remove primer-specific sequences and tagged with the IonCode Barcode Adapters. Finally, the amplified products were purified by performing a two-step cleanup using the Agencourt AMPure XP PCR purification system (Beckman Coulter, California, USA) at a bead to sample ratio of 1.15X and 1.0X, respectively. The Ion Library Equalizer Kit method was used to normalize the library concentration at ~100 pM. Finally, equal volumes of normalized DNA library were combined and amplified on Ion Sphere particles (ISP; ThermoFisher Scientific) by emulsion PCR using the Ion PI HiQ OT2 200 Kit (both ThermoFisher Scientific). Quality control was performed using the Ion Sphere Quality Control kit (ThermoFisher Scientific) to ensure that 10%–30% of template positive ISP were generated in the emulsion PCR. The template-positive Ion PI ISP were loaded on an Ion PI Chip and sequenced on an Ion S5^TM^ XLSequencer (ThermoFisher Scientific) with the Ion PI HiQ Sequencing 200 Kit (ThermoFisher Scientific) according to the manufacturer’s instructions.
